# *mcr*-Colistin Resistance Genes Mobilized by IncX4, IncHI2, and IncI2 Plasmids in *Escherichia coli* of Pigs and White Stork in Spain

**DOI:** 10.3389/fmicb.2019.03072

**Published:** 2020-01-17

**Authors:** Lourdes Migura-Garcia, Juan J. González-López, Jaime Martinez-Urtaza, J. R. Aguirre Sánchez, A. Moreno-Mingorance, A. Perez de Rozas, Ursula Höfle, Y. Ramiro, Narjol Gonzalez-Escalona

**Affiliations:** ^1^Centre de Recerca en Sanitat Animal, Institut de Recerca i Tecnologia Agroalimentàries, Barcelona, Spain; ^2^Research and Control of Emerging and Re-emerging Swine Diseases in Europe, OIE Collaborating Centre, CReSA, IRTA, Barcelona, Spain; ^3^Servei de Microbiologia, Hospital Universitari Vall d’Hebron, Universitat Autònoma de Barcelona, Barcelona, Spain; ^4^Centre for Environment, Fisheries and Aquaculture Science (CEFAS), Weymouth, United Kingdom; ^5^Centro de Investigación en Alimentación y Desarrollo, Culiacán, Mexico; ^6^Health and Biotechnology (SaBio) Group, Instituto de Investigación en Recursos Cinégéticos IREC (CSIC-UCLM-JCCM), Ciudad Real, Spain; ^7^Food and Drug Administration, Silver Spring, MD, United States

**Keywords:** *Escherichia coli*, colistin, *mcr*-plasmids, MinION nanopore, pigs, storks, Spain

## Abstract

Colistin has become the last-line antimicrobial for the treatment of multidrug resistant (MDR) *Enterobacterales* in human medicine. To date, several colistin resistance genes have been described. Of them *mcr*-1 is disseminated worldwide in *Escherichia coli* of human and animal origin. The aim of this study was to characterize *mcr*-mediated resistance plasmids from *E. coli* of animal origin in Spain. From our strain collection, 70 *E. coli* of pig origin collected between 2005 and 2014 (10 per year, except for years 2009–2010–2013) were randomly selected and screened for the presence of *mcr*-genes. Additionally, 20 *E. coli* isolated in 2011 from white storks (*Ciconia ciconia*) from the same urban household waste landfill associated colony were also included. Whole genome sequencing of *mcr*-positive isolates was carried out on a MiSeq (Illumina). Hybrid whole genome sequencing strategy combining nanopore and Illumina technologies were performed in a selection of isolates to close the genomes and plasmids and identify the presence of antimicrobial resistance genes. Minimum inhibitory concentration (MIC) was used to assess the susceptibility to colistin. Mating experiments were carried out to evaluate transferability of the *mcr*-genes. A total of 19 *mcr*-1 and one *mcr*-4 positive isolates were detected, 15 from pigs distributed during the study period, and five from storks collected in 2011. No other *mcr*-variants were found. The MICs for colistin ranged between 4 and >4 mg/L. High diversity of STs were detected among the *mcr-1* positive *E. coli* isolates, with only ST-10 shared between pigs and white storks. Except for one isolate, all were genotypic and phenotypically MDR, and five of them also harbored cephalosporin resistance genes (*bla*_CTX–M–__14_, *bla*_SHV–__12_, and three *bla*_CMY–__2_). *mcr*-1 genes were mobilizable by conjugation, associated with IncX4, IncHI2, and IncI2 plasmids. In our study, *mcr*-1 genes have been circulating in pig farms since 2005 harbored by a variety of *E. coli* clones. Its persistence may be driven by co-selection since plasmids containing *mcr*-1 also exhibit resistance to multiple drugs used in veterinary medicine. Furthermore, this is the first report of the presence of *mcr*-1 gene in isolates from white storks in Spain. This finding highlights the potential importance of wildlife that forage at urban household waste landfills in the transmission and spread of colistin resistance genes.

## Introduction

Antimicrobials have been used for many decades for the treatment of infectious diseases. This continued use, and in some cases, overuse has increased the emergence of resistant bacteria (Wall et al., 2016). The case of colistin is of particular interest. Colistin was introduced in human medicine in the early 1950s. Due to its toxicity when applied systemically, it was restricted to ophthalmic and topical use (Koch-Weser et al., 1970; Falagas et al., 2009). However, the emergence of pan-resistant enterobacteria causing infections in hospital settings has revived the use of colistin as the last-line treatment option. On the contrary, colistin has been prescribed in animal husbandry for the treatment of infections caused by *Enterobacterales* since the 1960s, especially in pig production. Furthermore, several studies carried in different European countries have identified the used of medicated feed mills supplemented with colistin for the prevention and/or treatment of post-weaning diarrhea (Trauffler et al., 2014; Cameron-Veas et al., 2016; Sjolund et al., 2016).

Resistance to colistin was attributed to chromosomal mutations that resulted in the modification in the lipid A of the LPS, reducing the binding of colistin (Rhouma et al., 2017). It was not until 2015 (Liu et al., 2016), when a plasmid-mediated mechanism of colistin resistance (*mcr*-1) was described for the first time, that the concern of its use reached the scientific community. The *mcr*-1 was first isolated in China from an *Escherichia coli* of pig origin and was harbored in a conjugative plasmid, facilitating its transfer to other enterobacteria. The potential spread of plasmid mediated *mcr*-genes from animals to humans rang the alarms of the Authorities, and the European Medicine Agency (EMA) reviewed the use of colistin in the European Union. Back in 2015, the use of colistin in swine production in Spain was estimated in 50 mg/PCU (population correction units) and a reduction target to 5 mg/PCU was set up for the next following years (EMA, 2016).

Currently, different variants of genes encoding colistin resistance have been identified (Xavier et al., 2016; Borowiak et al., 2017; Carattoli et al., 2017; Yin et al., 2017). However, *mcr*-1 has been isolated from enterobacteria of different origins, humans, livestock, wildlife, and the environment (Prim et al., 2016; Caltagirone et al., 2017; El Garch et al., 2018). Furthermore, in 90% of the cases, *mcr*-1 has been associated to IncX4, IncHI2, and IncI2 mobilizable plasmids (Matamoros et al., 2017; Elbediwi et al., 2019) facilitating its worldwide distribution (Li et al., 2017).

In addition, the emissions of antimicrobial resistant bacteria derived from human activities could have a negative effect in the environment and particularly in wildlife (Arnold et al., 2016). This is especially true for species, such as the white stork (*Ciconia ciconia*), that have adapted to using solid urban waste landfills as a reliable continuous food source. Spain holds the largest breeding population of storks in Europe, and due to availability of waste has also become a wintering area for storks from northern and central Europe. During the nesting period, storks are spatially bound to the surrounding areas. In some cases, these areas are waste landfills that represent a source of contamination (Gomez et al., 2016).

The aim of this study was to characterize the mechanisms involved in conferring resistance to colistin in isolates of animal origin in Spain. In particular, *E. coli* causing post-weaning diarrhea between consecutive batches of animals in pig farms, and in commensal *E. coli* isolated from white storks during the breeding season. Additionally, combining Illumina and Nanopore technology, we have completely sequenced the chromosome and closed whole plasmids of a set of isolates annotating all the genes, including those associated with antimicrobial resistance.

## Materials and Methods

### Study Design

From our strain collection, 70 *E. coli* of pig origin were randomly selected for screening the presence of colistin resistance genes. For each year, they represented 10 isolates obtained from diagnostic samples of post-weaning diarrhea collected from five different farms in consecutive batches of animals (one isolate per batch) between 2005 and 2014 (10 per year). Isolates from years 2009, 2010, and 2013 could not be found. Additionally, 20 *E. coli* obtained from white storks (*C. ciconia*) in 2011 from the same urban household waste landfill associated colony were also screened for the presence of *mcr*-genes. They represented fecal droppings collected at the side of different nesting platforms within the same colony. Ten of them were isolated in MacConkey agar and the other ten in MacConkey agar supplemented with cefotaxime (4 mg/L).

This study uses strains obtained from fecal samples of pigs with post-weaning diarrhea collected by the farm veterinarian and sent for diagnostics to our laboratory. Handling and sampling of the storks was carried out following all applicable international, national, and/or institutional guidelines for the care and ethical use of animals, specifically directive 2010/63/EU and Spanish laws 9/2003 and 32/2007, and Royal decrees 178/2004 and 1201/2005.

### Detection of *mcr* Genes

All isolates were tested by PCR methods for the presence of the *mcr*-1, *mcr*-2, *mcr*-3, *mcr*-4, and *mcr*-5 genes as previously described (Rebelo et al., 2018). No other mechanisms of resistance were tested.

### Antimicrobial Susceptibility Testing

All *mcr*-positive isolates were susceptibility tested using a minimum inhibitory concentration (MIC) broth microdilution (VetMIC GN-mo, National Veterinary Institute, Uppsala, Sweden). Antimicrobials tested were ampicillin (1–128 mg/L), cefotaxime (0.016–2 mg/L), ceftazidime (0.25–16 mg/L), nalidixic acid (1–128 mg/L), ciprofloxacin (0.008–1 mg/L), gentamicin (0.12–16 mg/L), streptomycin (2–256 mg/L), kanamycin (8– to 16 mg/L), chloramphenicol (2–64 mg/L), florfenicol (4–32 mg/L), trimethoprim (1–128 mg/L), sulfamethoxazole (8–1,024 mg/L), tetracycline (1–128 mg/L), and colistin (0.5–4 mg/L). Isolates were considered to be susceptible or resistant based on epidemiological cut-off values defined by EUCAST^1^.

### Mating Experiments

To evaluate the transferability of the genes conferring resistant to colistin, filter-mating experiments were performed with all isolates as donors and *E. coli* HB101 rifampicin resistant as recipient. Conjugations were carried out as described before (Bielak et al., 2011). Briefly, 500 uL of the mixture of each donor and recipient were placed in a sterile paper filter on a blood agar plate. After overnight incubation, the filter was diluted in PBS and 100 uL were spread on LB agar plates containing rifampicin (100 mg/L) and colistin (2 mg/L).

### Whole Genome Sequencing, Contigs Assembly and Annotation

*mcr*-positive isolates were selected for WGS studies. DNA was extracted using QIAGEN DNeasy^®^ Ultraclean Microbial Kit (Germany) following the manufacture’s recommendations. Short sequencing reads for each strain was generated using MiSeq Illumina sequencing with the MiSeq V3 kit using 2 × 250 base pair paired-end chemistry (Illumina, San Diego, CA, United States) according to manufacturer’s instructions, at 160–180× coverage. The libraries for the MiSeq were constructed using 100 ng of genomic DNA using Nextera DNA Flex kit (Illumina), according to manufacturer’s instructions. The genomes for each strain were *de novo* assembled using CLC Genomics Workbench v9.5.2 (QIAGEN) using defaults settings except that the minimum contig size threshold was set to 500 bp in length.

Based on results from the MiSeq assemble, five isolates harboring *mcr*-1 and IncHI2 replicons in the same contig, and therefore, with the two genes presumably located in the same plasmid were selected for further studies. They represented isolates of different origin collected during the study period and with the same replicon family. The whole genomes were sequenced and closed by using a combination of long reads and the short reads generated earlier. The long reads sequences for each strain were generated through nanopore sequencing using a MinION device (Nanopore, Oxford, United Kingdom). The sequencing libraries were prepared using the rapid barcoding sequencing kit RBK004 and run in a FLO-MIN106 (R9.4.1) flow cell, according to manufacturer’s instructions (Nanopore), for 48 h, at 50–130× average coverage. The sequencing library contained DNA fragmented randomly by a transposase present in the Fragmentation Mix of the RBK004 kit, rendering fragments >30 kb. The run was base called live using default settings (Minknow v18.05.5, Albacore). The genomes for each strain were obtained by *de novo* assembly, using Nanopore data and default settings within CANU program v1.6 (Koren et al., 2017).

A second assembly was generated using a SPAdes v3.11.1 (Bankevich et al., 2012) hybrid assembly (with default settings) using both Nanopore and MiSeq data generated for each strain. The final assembly (FA) was performed by comparing the SPAdes hybrid and CANU assemblies using Mauve (Darling et al., 2004) and filling in the missing regions in the SPAdes assembly with the CANU assembly. The FA sequences were annotated using the NCBI Prokaryotic Genomes Automatic Annotation Pipeline (PGAAP)^2^ (Tatusova et al., 2016). Antimicrobial resistance genes were annotated using the Comprehensive Antibiotic Resistance Database (CARD v3) (Jia et al., 2017). Plasmid identification was carried out using PlasmidFinder 2.1 (Carattoli et al., 2014).

We performed a Basic Local Alignment Search Tool (BLAST) (Altschul et al., 1990) to detect high similar published plasmids to pGN-295 sequenced herein, and we included pSLK172-1 (CP017632.1), pRDB9 (MH924589), pS38 (KX129782.1), 180-PT54 (CP015833.1), pCHL5009T-88k (CP032939.1), and NRZ14408 (LT599829). We used BLAST Ring Generator (BRIG) to display circular comparisons between the 11 *E. coli* plasmids (five identified in this study and six downloaded from NCBI with high similarity to our closed plasmids). For this purpose, plasmid pGN-295 was used as reference with an upper identity threshold of 90% and a lower identity threshold of 70% and a nucleotide search using BLASTn (Supplementary Table 3).

### Nucleotide Sequence Accession Numbers

The draft genome sequences of the 20 *E. coli* strains used in our study are available in GenBank under the accession numbers listed in the Supplementary Table 1.

### Phylotyping and Phylogenetic Analyses

The Clermont et al. (2013) phylotyping scheme was performed by multiplex PCR for detecting phylotypes A, B1, B2, C, D, and F as described before (Clermont et al., 2013).

The initial identification of the strains was performed using an *in silico E. coli* MLST approach, based on the information available at EnteroBase for *E. coli* website^3^ and using Ridom SeqSphere + software v2.4.0 (Münster, Germany)^4^ to perform the *in silico* search. Seven housekeeping genes (*dnaE*, *gyrB*, *recA*, *dtdS*, *pntA*, *pyrC*, and *tnaA*), described previously for *E. coli* were used for determining the STs (Wirth et al., 2006).

The phylogenetic relationship of the strains was assessed by a custom core genome multilocus sequence typing (cgMLST) analysis using Ridom SeqSphere + software v2.4.0. We used the cgMLST scheme reported earlier (Lorenz et al., 2017; Gonzalez-Escalona and Kase, 2019). The genome of O157:H7 strain Sakai (NC_002695.1) was used as the reference for the cgMLST. A total of 4,651 genes were used as templates for the analysis of the *E. coli* strains from this study. We also added nine other genomes of known *E. coli* strains to establish their phylogenetic context (Supplementary Table 2). A Neighbor-Joining (NJ) tree using the appropriate genetic distances was built after the cgMLST analysis.

### *In silico* Virulence Genes Detection

Each *de novo* assembled genome was screened *in silico* for the presence of 102 virulence genes reported for *E. coli* as described elsewhere (Gonzalez-Escalona and Kase, 2019).

## Results

### Presence of *mcr*-Genes

Out of 70 *E. coli* isolates of pig origin, 14 were positive for the presence of the *mcr*-1. Additionally one isolate collected in 2007 was positive for *mcr*-4.2. They were distributed between the study-period (Table 1). No other *mcr*-variants were detected. Additionally, five isolates of white stork origin obtained from 2011 were also positive, four contained *mcr*-1, and one contained the *mcr*-1.2 variant.

**TABLE 1:** Resistance genes for the different antimicrobials families found in isolates bearing *mcr*-plasmids.

**ID**	**Year**	**Origin**	**Colistin**	**Aminoglycoside**	**Sulfonamide**	**Trimethoprim**	**Tetracycline**	**Phenicol**	**Beta-lactam**	**MLS**
GN-295	2005	Pig	*mcr*-1.1	*aac*(3)-IIa, *aadA1*, *aadA2*, *strA*, *strB*	*sul1*, *sul2*, *sul3*	*dfrA1*	*tet*(A)	*catA1*, *cml*, *cmlA1*	TEM-1A	
GN-444	2005		*mcr*-1.1	*aac(3)-IIa*, *aac(3)-IVa*, *aadA1*, *aadA2*, *aph(3′)-Ia*, *aph(4)-Ia*, *strA*, *strB*	*sul2*, *sul3*	*dfrA12*	*tet*(B), *tet*(M)	*catA1*, *cmlA1*, *floR*	TEM-1A	*mef*(B)
GN-445	2005		*mcr*-1.1	*aac(3)-IIa*, *aac(3)-IVa*, *aadA1*, *aadA2*, *aph(3′)-Ia*, *aph(4)-Ia*, *strA*, *strB*	*sul2*, *sul3*	*dfrA12*	*tet*(B), *tet*(M)	*catA1*, *cmlA1*, *floR*	TEM-1A	*mef*(B)
GN-609	2006		*mcr*-1.1	*aac(3)-IVa*, *aadA1*, *aph(4)-Ia*, *strA*, *strB*	*sul1*, *sul2*	*dfrA1*	*tet(A)*, *tet(M)*		TEM-1A	*mph*(B)
GN-1035	2007		*mcr*-1.1	*aadA1*, *aadA2*, *aph(3′)-Ia*	*sul1*, *sul3*	*dfrA1*	*tet*(A)	*cmlA1*	TEM-1B	
GN-1036	2007		*mcr*-1.1	*aac(3)-IIa*, *aadA1*, *aadA2*, *aph(3′)-Ic*, *strA*, *strB*	*sul1*, *sul2*, *sul3*	*dfrA1*	*tet*(A)	*catA1*, *cml*, *cmlA1*	TEM-1A	
GN-1044	2007		*mcr*-1.1	*aac(3)-IIa*, *aadA1*, *aadA2*, *aph(3′)-Ia*, *strA*, *strB*	*sul1*, *sul2*, *sul3*	*dfrA1*	*tet*(A), *tet*(B)	*catA1*, *cml*, *cmlA1*	TEM-1A	*Inu*(G), *mdf*(A)
GN-1058	2007		*mcr*-4.2	*aac(3)-IId*, *aac(3)-IVa*, *aadA1*, *aadA2*, *aph(4)-Ia*, *strA*, *strB*	*sul3*	*dfrA12*	*tet*(A), *tet*(M)	*catA2*, *cmlA1*	TEM-1B	*lnu*(F)
GN-1639	2008		*mcr*-1.1	*aac(3)-IVa*, *aadA1*, *aadA2*, *aph(4)-Ia*, *strA*, *strB*	*sul3*	*dfrA12*	*tet*(A), *tet*(M)	*cmlA1*, *floR*	TEM-1B	
GN-2933	2011		*mcr*-1.1				*tet*(A)			
GN-3020	2012		*mcr*-1.1	*aac(3)-IVa*, *aadA1*, *aadA2*, *aph(4)-Ia*, *strA*, *strB*	*sul3*	*dfrA12*	*tet*(C)	*cmlA1*, *floR*	TEM-1B	
GN-3021	2012		*mcr*-1.1	*aac(3)-IVa*, *aadA1*, *aadA2*, *aph(4)-Ia*, *strA*, *strB*	*sul3*	*dfrA12*	*tet*(C)	*cmlA1*, *floR*	TEM-1B	
GN-3022	2012		*mcr*-1.1	*aadA1*, *aadA1*, *aph(3′)-Ic*, *aac(6′)Ib-cr*	*sul1*	*dfrA1*	*tet*(B), *tet*(M)	*catA1*, *catB3*	TEM-1B	*mph*(E), *msr*(E)
GN-102A	2014		*mcr*-1.1	*aac(3)-IVa*, *aadA1*, *aph(3′)-Ia*, *aph(4)-Ia*, *strA*, *strB*	*sul1*, *sul2*, *sul3*	*dfrA1*, *dfrA5*	*tet*(A)	*floR*	TEM-1B, CTX-M-14	
GN-115A	2014		*mcr*-1.1	*aadA1*, *aadA2*	*sul3*	*dfrA1*	*tet*(A)	*catA1*, *floR*	SHV-12	
171	2011	Stork	*mcr*-1.1				*tet*(A)		TEM-1B	
172	2011		*mcr*-1.1	*aac(3)-IId*, *aadA2*, *strA*, *strB*	*sul1*, *sul2*	*dfrA12*	*tet*(A)	*catA1*	TEM-1B	*mph*(A)
173	2011		*mcr*-1.1				*tet*(A)		TEM-1B, CMY-2	
176	2011		*mcr*-1.2	*aac(3)-Iva*, *aadA1*, *strB*, *aph(4)-Ia*,	*sul3*	*dfrA1*	tet(B)	catA1	TEM-1B, CMY-2	
178	2011		*mcr*-1.1	*aac(3)-IIa*, *aadA1*, *aadA2*, *aph(3′)-Ic*, *strA*, *strB*	*sul1*, *sul2*, *sul3*	*dfrA1*, *dfrA12*	tet(A)	catA1, cmlA1	TEM-1A, TEM-1B, CMY-2	

* In gray isolates belonging to the same farm, but consecutive batches. MLS: Macrolides, lincosamides, streptogramines, ID: Identification.*

### Antimicrobial Susceptibility Testing and Presence of Resistance Genes

The 20 isolates exhibited a MIC to colistin ≥4 mg/L. All *E. coli* were phenotypically resistant to tetracycline confirmed by the presence of *tet*(A), *tet*(B), *tet*(C), *tet*(M) or the combination of some of these genes, and except for one isolate of pig origin, they were also resistant to ampicillin (Table 1) conferred by TEM-1A or TEM-1B. Furthermore, co-resistance was observed for streptomycin, kanamycin and sulfonamides (67%) with an average of six different genes coding resistance for aminoglycosides. Sixteen isolates exhibited resistance to florfenicol conferred by the presence of a unique gene or the combination of different genes. Nalidixic acid and ciprofloxacin resistance was detected in 14 isolates (56%). The presence of single point mutations in quinolone resistance-determining regions (QRDR) in chromosomal *gyrA*, *parC*, or *parE* genes was confirmed for these isolates. Plasmid mediated quinolone resistance genes were not detected. Except for isolate GN-2933, they were all multidrug resistant (MDR) (phenotypically resistant to at least three different families of antimicrobials). Phenotypic results correlated with the presence of the different resistance genes for each antimicrobial family. As illustrated in Table 1, the number of resistance genes varied depending of the isolate. Resistance to cephalosporins coded by CTX-M-14 and SHV-12 was observed in two isolates of pig origin and was confirmed by the MIC results (MICs for cefotaxime and ceftazidime >2 and >16 mg/L, respectively). CMY-2 was confirmed in three isolates of white stork origin.

### Characterization of *E. coli* Bearing *mcr*-1 Plasmids

Among pig isolates, phylotyping identified seven isolates belonging to group A, five to group B1, two to group D and one to E. *E. coli* of white stork origin belonged to group A (*n* = 3), B1 (*n* = 1), and E (*n* = 1). B2 was never detected (Table 2).

**TABLE 2:** Serotype, phylogroup, MLST, plasmid replicon associated to *mcr*-genes, flanking regions and virulence genes.

**ID**	**Origin**	**Serotype**	**phylo**	**MLST, CC**	**mcr-plasmid**	**size**	**GC content**	**ORF**	***mcr*-1 context**	**Virulence genes**
GN-295	Pig	Ounk:H32	A	ST-10, CC10	IncHI2	356,700	47.2	447	*ISApl1*-*mcr*-1-hp-*ISApl1*	*gad*, *iss*
GN-444		O25:H28	B1	ST-156, CC156	IncX4	Unknown			hp-*mcr*-1-hp	*gad*, *iss*, *lpfA*
GN-445		O25:H28	B1	ST-156, CC156	IncX4	Unknown			hp-*mcr*-1-hp	*gad*, *iss*, *lpfA*
GN-609		O98:H12	A	ST-10, CC10	IncX4	Unknown			hp-*mcr*-1-hp	*aaiC*, *capU*, *celb*, *gad*, *iha*, *iss*, *katP*
GN-1035		O15:H45	E	ST-118	IncX4	Unknown			hp-*mcr*-1-hp	*air*, *astA*, *celb*, *eilA*, *gad*, *stb*
GN-1036		O8:H20	A	ST-3205	IncHI2	265,340	47.2	344	*ISApl1*-*mcr*-1-hp-*ISApl1*	*astA*, *capU*, *gad, stb*
GN-1044		O138:H	A	ST-100, CC165	IncHI2	Unknown			*ISApl1*-hp-*mcr*-1-hp	*astA*, *capU*, *gad*, *iha*, *K88ab*, *ltcA*, *stb*
GN-1058		O123:H23	B1	ST-224	IncX4	Unknown			*mcr*-4.2-hp-*relE*	*astA*, *gad*, *lpfA*
GN-1639		O20:H20	A	ST-542	IncX4	Unknown			hp-*mcr*-1-hp	*astA*, *gad*
GN-2933		O157:H19	B1	ST-763	IncI2	Unknown			hp-*mcr*-1-hp	*astA*, *gad*, *lpfA*, *stb*
GN-3020		O138:H14	D	ST-42	IncX4	Unknown			hp-*mcr*-1-hp	*air*, *astA*, *cba*, *cma*, *fedA*, *fedF*, *iha*, *iss*, *lpfA*, *ltcA*, *sta1*, *stb*
GN-3021		O138:H14	D	ST-42	IncX4	Unknown			hp-*mcr*-1-hp	*air*, *astA*, *cba*, *cma*, *fedA*, *fedF*, *iha*, *iss*, *lpfA*, *ltcA*, *sta1*, *stb*
GN-3022		O29:H51	B1	ST-156, CC156	IncX4	Unknown			hp-*mcr*-1-hp2	*gad*, *iss*, *lpfA*, *tsh*
GN-102A		O11:H8	A	ST-448, CC488	IncHI2	Unknown			hp-hha-*mcr*-1-hp	*celb*, *gad*, *iroN*, *iss*, *lpfA*, *mchF*
GN-115A		O98:H12	A	ST-10, CC10	IncHI2	252,383	46.1	319	ISApl1-*mcr*-1-hp	*aaiC*, *capU*, *celb*, *gad*, *iha*, *iss*, *katP*
171	Stork	O5:H4	A	ST-93, CC168	IncX4	Unknown			hp-*mcr*-1-hp	*capU*, *cma*, *gad*, *iroN*, *iss*, *tsh*
172		O86:H51	E	ST-1011	IncX4	Unknown			hp-*mcr*-1-hp	*eilA*, *gad*, *iss*
173		O5:H4	A	ST-93, CC168	IncX4	Unknown			hp-*mcr*-1-hp	*capU*, *cma*, *gad*, *iroN*, *iss*, *tsh*
176		O107:H10	A	ST-10, CC10	IncHI2	235,260	46.1	309	putative kinase-*mcr*-1.2-hp	*gad*, *lpfA*, *astA*
178		O18ac:H7	B1	ST-351	IncHI2	264,642	47.2	344	*ISApl1*-*mcr*-1-hp-*ISApl1*	*cma*, *gad*, *iroN*, *iss*, *lpfA*

*In gray isolates belonging to the same farm, but consecutive batches.*

WGS identified a high variety of serotypes among the different isolates, with five of them of pig origin typically associated with post-weaning diarrhea, three of serotype O138, one O8, O15, and O157.

**FIGURE 1:**
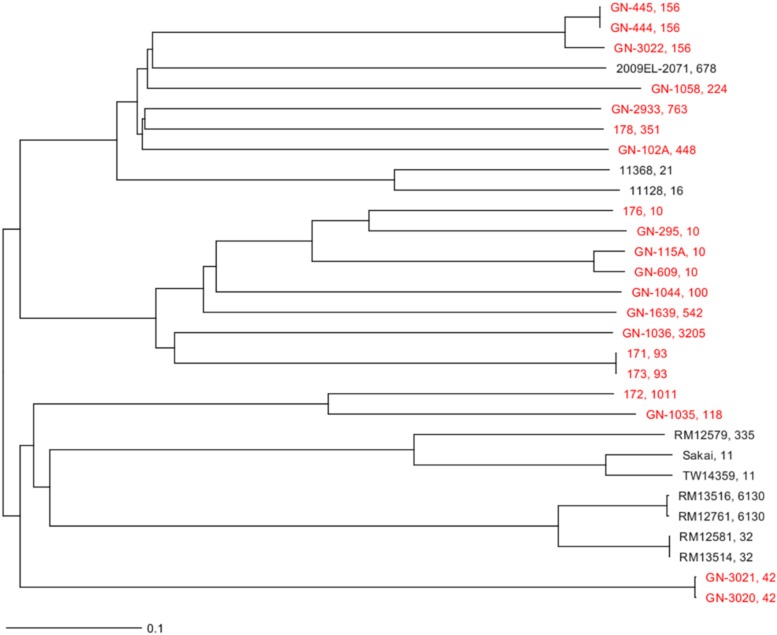
Neighbor joining phylogenetic tree generated by a cgMLST analysis of the strains sequenced in this study and 10 other known *E. coli* strains (Supplementary Table 2). The genome of *E. coli* O157:H7 strain Sakai (NC_002695) was used as reference. The final cgMLST analysis was based on 2514 shared loci among those strains. The sequenced strains in this study are in red. Next to the name of the strain is their ST separated by a comma.

There was a polyclonal expansion of *E. coli* bearing *mcr*-1 plasmids, with 20 isolates belonging to 14 different MLST types. Some of these ST types, such as ST42, ST118, ST3205, ST763, and ST351 are not very frequent according to the MLST database (see text footnote 3). ST10 was detected in three isolates of pig origin distributed over the years. This was the only ST type shared between *E. coli* of pig and white stork origin. Phylogenetic studies demonstrated the presence of the same clonal linages of *E. coli* causing post-weaning diarrhea between different batches of animals in two different occasions; isolates GN-444 and GN-445 in 2005 and GN-3020 and GN-3021 in 2012 (Table 2). Additionally, two isolates obtained from white stork droppings (171 and 173) were clonally related (Figure 1).

WGS also detected a wide variety of virulence genes, ranging from 2 to 12 (Table 2), with no apparent association between number of virulence genes, phylogroup or ST type.

### Plasmid Characterization and Genetic Context of the *mcr*-Genes

All *mcr*-plasmids (*mcr*-1 and *mcr*-4) successfully transferred the colistin resistant gene by conjugation to the recipient strain, demonstrating their ability to move the *mcr*-genes between isolates.

For all isolates, Illumina sequencing allowed to detect *mcr*-1 genes in the same contigs as replicons of the families IncX4 (*n* = 12), IncHI2 (*n* = 7), and IncI2 (*n* = 1). In the case of *mcr*-4.2, it was also associated to IncX4 (Table 2). Additionally, isolates contained a wide variety of replicons of different incompatibility groups presumably in different plasmids, ranging from 3 to 12. IncF and ColRNAI were the more represented (*n* = 15) followed by IncFIB (*n* = 11), IncI1 (*n* = 10), Col(MG8282) (*n* = 9) and finally, IncHI2A, IncHI2, and col156 (*n* = 8, for each of them).

**FIGURE 2:**
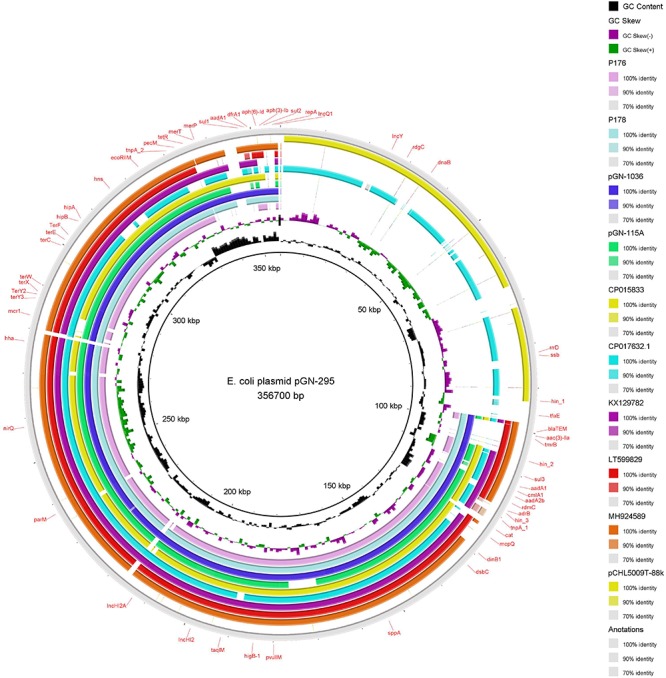
BRIG visualization of multiple *E. coli* plasmids comparisons. The solid innermost ring (black) correspond to the reference plasmid (pGN-295). After this ring plasmid p176 is shown (light purple). The third ring correspond to p178 (light blue), then pGN-1036 (navy blue) and pGN-115A (green). Additionally high identity plasmids to pGN-295 were download and added to the comparisons. The fifth (mustard) correspond to CP015833 plasmid, next CP07632.1 (aqua), KX129782 (purple), LT599829 (red), MH924589 (brown), pCHL5009T-88k (yellow), and finally we add the reference again (gray). The label in the outer ring represent the annotation on the genes associated to virulence, antibiotic resistance, stress resistance and replicons.

The sizes of the five IncHI2 plasmids sequenced in this study varied between 235,260 and 356,700 bp (Table 2) and were all pMLST ST-4. In general, the architecture of the IncHI2 plasmids appeared to present high level of similarity with all of them having the conjugative transfer system, HigB-HigA toxin-antitoxin system for plasmid maintenance, macrolide resistance efflux pump *mef*(B) and a tellurium resistant operon (Figure 2). Except for p176, they also harbored a mercury resistance operon, which also included the genes for cobalt-zinc-cadmium resistance. In particular, pGN-115A contained the copper resistance gene *pcoE*. Plasmid p176 also possessed a MazEF toxin-antitoxin system. Additionally, these high molecular weight plasmids also yielded resistance genes for different families of antimicrobials, including aminoglycosides, beta-lactams, tetracycline, phenicols, sulfonamides, and trimethoprim (Table 2). Interestingly, pGN-295 with the largest molecular weight, presented and extra-DNA region highly similar to a previously sequenced plasmid named pCHL5009T-88k and detected in and *E. coli* of human origin in New Zealand (*E. coli* NRZ14408). In that region, an IncY replicon was detected.

Furthermore, there were three different *mcr*-1 context or arrangements found in all these families of plasmids (Table 2). *mcr*-1 was flanked upstream and downstream by the *ISApl1* elements, loss of the downstream *ISApl1* element, and the complete loss of *ISApl1* elements surrounding the *mcr*-1. Regarding *mcr*-4.2, following the gene was an ORF coding for a hypothetical protein and a *relA* gene.

## Discussion

Plasmid mediated colistin resistance has been described worldwide in a variety of *Enterobacterales* of different origins, environment, food producing animals, wildlife and humans (Zurfluh et al., 2016; Guenther et al., 2017; Ovejero et al., 2017; Barlaam et al., 2019; Elbediwi et al., 2019; Lalaoui et al., 2019; Mendes Oliveira et al., 2019; Nang et al., 2019; Zając et al., 2019). In particular, in Spain, *mcr*-1 has been described circulating in pig farms for many years (Quesada et al., 2016; Garcia-Menino et al., 2018), and our results demonstrate its presence in pig farms in Spain as early as 2005. Although other *mcr*-gene variants have also been reported in Spain (Carattoli et al., 2017), studies by Garcia-Menino et al. (2018) screening large number of *E. coli* isolates from diagnostic cases of post-weaning diarrhea detected *mcr*-1 as the most common variant followed by *mcr*-4. Our study also confirmed similar results, although more recently described *mcr*-genes have not been tested herein. Furthermore, in two different occasions, carry over of the same clones causing diarrhea between batches of animals within the same farm was observed. In addition to the consumption of antimicrobials, internal biosecurity including cleaning and disinfection can play an important role in the maintenance of antimicrobial resistant bacteria in the farms (Raasch et al., 2018), facilitating its transmission and persistence between different batches of animals. However, a vertical transmission from sows to their litter cannot be disregarded.

Although we do not have antimicrobial consumption data for these farms, it is interesting to notice that in pig production in Spain, before the finding of the *mcr*-genes, colistin was the first choice antimicrobial for the treatment of *E. coli* causing post-weaning diarrhea (Moreno, 2014). The majority of these treatments were prescribed empirically instead of based on antimicrobial susceptibility testing. This fact highlights the importance of performing phenotypic tests to provide a successful therapy for the treatment of disease and to acquire epidemiological surveillance data of the phenotypes circulating in the farms. Generally, National Programs in Europe only include commensal *E. coli* to monitor antimicrobial resistance from food producing animals (Garcia-Migura et al., 2014), and little is known about epidemiology and resistant mechanisms present in diagnostic samples. Additionally, except for one, all of the isolates herein were MDR, impairing a successful treatment of post-weaning diarrhea and most probable facilitating the proliferation of the pathogen, since the antimicrobials would eliminate beneficial microbiota that could colonize the same niche and compete for the nutrients (Soler et al., 2018).

Wildlife is generally not treated with antibiotics but has been shown to acquire commensal microbiota carrying resistance genes when in contact with anthropized environments and livestock (Gomez et al., 2016; Darwich et al., 2019). To the best of our knowledge, this would be the first report of ST10 *E. coli* isolates *mcr*-1 positive described in white storks. The colony from which these originated is located on the premises of a solid urban waste disposal site. Acquisition of these genes is probably through foraging of feed at the landfill. Several studies have reported the presence of *mcr*-1 in migratory birds (Liakopoulos et al., 2016; Mohsin et al., 2016; Ruzauskas and Vaskeviciute, 2016; Tarabai et al., 2019), including co-resistance to colistin and cephalosporins. In fact, three of our isolates of white stork origin contained *mcr*-1 and *bla*_CMY–__2_ genes within the same host. The fact that a large proportion of the European white stork population is migratory may facilitate the spread of the *mcr*-1 gene between different continents and emphasizes the importance of studies monitoring wildlife as potential reservoirs of resistance genes with an impact in public health along their migration routes. Furthermore, as especially juvenile storks with different geographical origins may share the same areas during wintering in Africa, this could potentially lead to cross contamination between storks from different regions (Flack et al., 2016).

The diversity of clones in terms of serotype, phylotype and ST type encountered among isolates of both, pigs and white stork origin was very high, demonstrating a wide range of hosts harboring *mcr*-plasmids. The unique ST type shared between isolates of both origins was ST10. ST10 is widely disseminated in humans and animals and frequently associated not only with the presence of cephalosporin resistance genes (Ojer-Usoz et al., 2017), but currently also with the occurrence of *mcr*-1 (Matamoros et al., 2017; Elbediwi et al., 2019). In fact, two ST10 isolates of pig and white stork origin from our study exhibited co-resistance to colistin and cephalosporins, both critically important antimicrobials to treat severe human infections caused by MDR Gram-negative in hospital settings (World Health Organization [WHO], 2017). Furthermore, ST10 has been identified as a high-risk lineage causing human extraintestinal infections (Manges et al., 2019). In Spain *mcr*-1 ST10 has also been described in clinical isolates causing severe infections (Lalaoui et al., 2019). The fact that food-producing animals, and wildlife associated to human settlements such as white storks that frequently nest on structures within villages and cities, could be a reservoir and source of potential pathogens with a MDR profile is worrisome, and emphasizes the need to coordinate efforts from human and veterinary sectors within a One Health approach.

Despite the clonal diversity observed among *E. coli* isolates in this study, only three plasmid incompatibility groups, IncX4, IncHI2, and IncI2 could be directly associated to the presence of the *mcr*-1 gene. These replicons are well described in the literature for their fidelity toward *mcr*-1 genes (Matamoros et al., 2017; Sun et al., 2017). Zurfluh et al. (2017) described IncI2 *mcr*-1 positive plasmids carried by *E. coli* strains belonging to distinct STs than the one in our study, confirming the high transmissibility of plasmids containing *mcr*-1 gene. Interestingly, the BLAST of our sequenced IncHI2 plasmids, retrieved similar backbones of small molecular weight plasmids with the absence of *mcr*-1. Furthermore, a region of pGN-295 containing an IncY replicon matched a plasmid obtained from a patient in New Zealand, pCHL5009T-88k (CP032939.1). It is impossible to find an epidemiological connection between plasmids found so far apart. However, these results suggest that some plasmids might be highly adapted to their host and able to evolve through recombination processes. They are dynamic and plastic structures prone to merge and generate mega-plasmids, providing an advantage for the bacterium.

Mating experiments demonstrated the capability of all the isolates to mobilize the resistant gene to a recipient strain, and WGS data corroborated the presence of the conjugative elements. It is worth noticing that closed IncHI2 plasmids from pigs and white storks also carried a variety of resistance genes for different families of antimicrobials (aminoglycosides, beta-lactams, tetracycline, sulfonamides, phenicols, and trimethoprim). This means that even withdrawing the use of colistin in pig farms, other common antimicrobials used during the rearing cycle in pig production such as tetracycline or beta-lactams could co-select for the presence of colistin resistance, persisting for long periods in food producing animals. Interestingly, these five IncHI2-ST4 plasmids also contained genes encoding resistance for heavy metals (mercury, cobalt, zinc, cadmium, copper and tellurium) and small multidrug resistance efflux transporter (QacE) conferring resistance to quaternary ammonium compounds (QAC). QAC have been commonly used as disinfectants with different applications in health care and the food-industry (Kaskova et al., 2007; Quinn et al., 2015). Resistance to disinfectants presumably confers these clones the capacity to survive under extreme conditions. Furthermore, since mazEF and hipBA toxin–antitoxin systems were detected, these isolates are bound to the plasmid, and losing the plasmid will trigger the pathway of cellular death (Engelberg-Kulka et al., 2005). Strategies to target these systems appear to be a promising therapy to defeat these MDR pathogens due to the bactericidal effect (Rownicki et al., 2018).

The *mcr*-1 flanking areas described herein have also been reported in studies elsewhere for the three-replicon families (Hadjadj et al., 2017; Zurfluh et al., 2017; Duggett et al., 2018; Zając et al., 2019). The *ISApl1* flanking the *mcr*-1 gene appears to be a very efficient mechanism for “traveling” around, facilitating *mcr*-1 transposition between a limited number of incompatibility families of plasmids (Matamoros et al., 2017). Same genetic context has been identified in IncHI2 plasmids herein together with the loss of the *ISApl1* element downstream the *mcr*-1 gene or the total absence of *ISApl1*, anchoring the *mcr*-1 gene to the plasmid. These differences in the surrounding regions of *mcr*-1 probably indicate different stages in the evolution of the plasmid once integrating the transposon, becoming more stable while losing the *ISApl1* elements (Wang et al., 2018). In the case of *mcr*-4, recent studies have also detected similar flanking regions to the one described here in *E. coli* causing post-weaning diarrhea presumably in Italy (MG800338). Again, it is difficult to elucidate an epidemiological relation, unless providers of animals were shared at some point between European countries. More likely, *mcr*-genes have found a successful plasmid expansion by transposition and conjugation to perpetuate its transmission.

To conclude, we confirmed the polyclonal expansion of IncX4 and IncHI2 plasmids bearing *mcr*-1 in *E. coli* causing post-weaning diarrhea in pig farms in Spain since at least 2005. This highlights the role of food producing animals as reservoirs of antimicrobial resistant genes with impact in human health. Additionally, to the best of our knowledge this is the first report of *mcr*-1 detected in commensal *E. coli* isolated from white stork as far back as 2011. Anthropogenic pressure is highly associated to the presence of these resistance mechanisms. The study also showed the benefits of applying an advanced strategy combining Illumina and Nanopore sequencing technologies to close the genomes and plasmids as a key element to gain a basic understanding on genetic variation and dynamics of plasmid transfer. Using this innovative approach along with comprehensive analysis within a One Health context, we might be able to design strategies to minimize the emergence and persistence of resistance mechanisms within food producing animals and improve our knowledge about the spread of these mechanisms driven by forces such as migratory movements of wild birds. Hence, efforts should be made to mitigate the effect of the anthropogenic pressure and the released of antimicrobial resistance genes into the environment.

## Data Availability Statement

The draft genome sequences of the 20 *E. coli* strains used in our study are available in GenBank under the accession numbers listed in the Supplementary Table 1.

## Ethics Statement

Ethical review and approval was not required for the animal study because this study is performed with *E. coli* of fecal samples of animal origin stored in our freezer.

## Author Contributions

LM-G designed the study. AP, YR, and UH contributed to the collection of samples. NG-E, JG-L, JM-U, JA, AM-M, and LM-G performed the sequencing, analyses, and interpretation. LM-G drafted the first manuscript. All authors contributed with the final writing, reviewing, and editing.

## Conflict of Interest

The authors declare that the research was conducted in the absence of any commercial or financial relationships that could be construed as a potential conflict of interest.

The handling Editor declared a shared affiliation, though no other collaboration, with the authors JG-L and AM-M at time of review.

## Footnotes

1 http://www.eucast.org/

2 http://www.ncbi.nlm.nih.gov/genome/annotation_prok

3 https://enterobase.warwick.ac.uk/species/index/ecoli

4 http://www.ridom.com/seqsphere
